# p53 targets TSPAN8 to prevent invasion in melanoma cells

**DOI:** 10.1038/oncsis.2017.11

**Published:** 2017-04-03

**Authors:** G Agaësse, L Barbollat-Boutrand, M El Kharbili, O Berthier-Vergnes, I Masse

**Affiliations:** 1 Université de Lyon, Lyon, France; 2 Université Lyon 1, Lyon, France; 3 CNRS, UMR5534, Centre de Génétique et de Physiologie Moléculaires et Cellulaires, Villeurbanne, France

## Abstract

Cutaneous melanoma is a very deadly cancer because of its proclivity to metastasize. Despite the recent development of targeted and immune therapies, patient survival remains low. It is therefore crucial to enhance understanding of the molecular mechanisms underlying invasion. We previously identified tetraspanin 8 (TSPAN8) as an important modulator of melanoma invasiveness, and several of its transcriptional regulators, which affect TSPAN8 expression during melanoma progression toward an invasive stage. This study found that TSPAN8 promoter contains consensus-binding sites for p53 transcription factor. We demonstrated that p53 silencing was sufficient to turn on Tspan8 expression in non-invasive melanoma cells and that p53 acts as a direct transcriptional repressor of TSPAN8. We also showed that p53 modulated matrigel invasion in melanoma cells in a TSPAN8-dependent manner. In conclusion, this study reveals p53 as a negative regulator of Tspan8 expression. As TP53 gene is rarely mutated in melanoma, it was hitherto poorly studied but its role in apoptosis and growth suppression in melanoma is increasingly becoming clear. The study highlights the importance of p53 as a regulator of melanoma invasion and the concept that reactivating p53 could provide a strategy for modulating not only proliferative but also invasive capacity in melanoma treatment.

## Introduction

Cutaneous melanoma is one of the leading causes of death worldwide because of its proclivity to metastasize. Immunotherapies and highly selective targeted therapies have led to promising clinical advances, but most patients relapse because of acquired treatment resistance. Consequently, combination strategies have recently been developed, with substantial improvement in clinical outcome. Nevertheless, there is an urgent need to discover new markers of melanoma progression as potential therapeutic targets. Therefore, understanding the molecular and cellular mechanisms underlying the initial steps of melanoma progression is crucial to identifying new therapeutic strategies that could be combined with current therapies to improve survival.

We previously identified tetraspanin 8 (Tspan8) protein as an important factor in early melanoma invasion.^1^ Tspan8 is 1 of the 33 mammalian members of the tetraspanin family, composed of transmembrane proteins that organize laterally, together or with other membrane partners such as integrins, to form ‘tetraspanin webs’. These platforms signal within cells to regulate many cellular processes: adhesion, migration, invasion or survival (for a review, see Yanez-Mo *et al.*^2^). Tspan8 has been implicated in many types of cancer. Overexpression was reported in glioma and colorectal, esophageal, hepatic, gastric and pancreatic carcinoma. Furthermore, Tspan8 expression correlates strongly with metastatic potential in liver, colon and pancreatic carcinoma.^3, 4, 5, 6, 7, 8, 9^ In colon and liver cancer, Tspan8 exerts a pro-invasive function by controlling cell–cell and cell–matrix interactions through its association with membrane partners such as α6β4 integrin-protein kinase C (PKC)-activated, E-cadherin, EpCAM, claudin-7 and CD44 (for a review, see Hemler^10^). Moreover, Tspan8 may be a promising new therapeutic target, as Tspan8-specific antibodies were shown to reduce cell motility, block tumor angiogenesis *in vivo* and inhibit the growth of colorectal tumors in a xenogeneic nude mouse model^7, 11, 12^ and significantly reduce incidence of epithelial ovarian cancer metastasis *in vivo*.^13^

In cutaneous melanoma, we previously showed that Tspan8 expression is undetectable in normal skin and becomes expressed at very high levels in primary melanomas and lymph node metastases. We also demonstrated that Tspan8 overexpression is sufficient to increase cell invasion capacity.^1^ Previous studies of Tspan8 focused on its expression level, functions and biochemical interactions with protein partners during cancer development, but nothing is known about its transcriptional regulation. To understand how Tspan8 expression is turned on, inducing invasive properties in melanoma cells, we recently performed an RNA interference-based screen, for several genes known to regulate the metastatic process. Several TSPAN8 transcriptional regulators were identified, including lung-cancer metastasis-related protein 1, which increases TSPAN8 expression and causes loss of melanoma cell–matrix adherence, leading to cell invasion.^14^

This study identified Tspan8 as a p53 transcriptional target for melanoma cell invasion, suggesting that p53, although rarely mutated but sometimes inactivated in melanoma, may have an important role not only in apoptosis and cell cycle arrest but also in melanoma cell invasion. TP53 has not been identified in our recent RNA interference screening because it was not included in the array of genes tested.^14^ However, we showed here that the promoting sequence of TSPAN8 contains consensus-binding sites for p53 transcription factor. We demonstrated that p53 silencing is sufficient to induce Tspan8 expression in melanoma cells. We also showed, by chromatin immunoprecipitation and luciferase assays, that p53 is a direct transcriptional repressor of Tspan8. We finally demonstrated that p53 inhibition led to an increase of matrigel invasion in a Tspan8-dependent manner. Overall, our data emphasize the crucial role that the p53 transcription factor has in melanoma aggressiveness via its involvement in regulating TSPAN8 expression.

## Results and Discussion

### The internal promoter of TSPAN8 contains consensus-binding sites for p53 transcription factor

**Figure 1:**
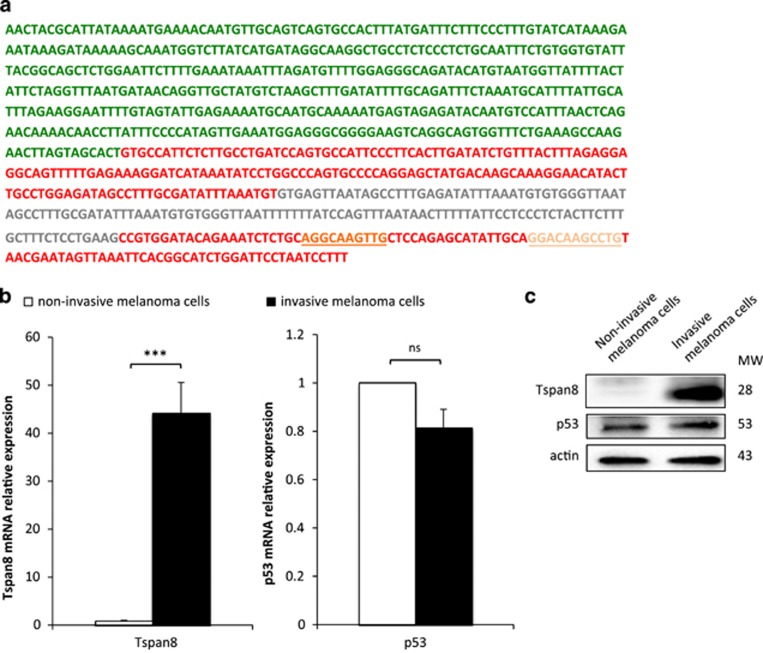
TSPAN8 functional promoter and p53 expression in melanoma cells. (**a**) Promoting sequence of the TSPAN8-002 isoform shows p53 consensus-binding sites as predicted by Genomatix GmbH, Munich, Germany. Promoter upstream of the transcriptional starting site (TSS) is colored in green, exons 1 and 2 in red and intron 1 in gray. The two putative half-sites for p53 binding are underlined, ‘site 1’ is colored in dark orange and ‘site 2’ in light orange. (**b**) Expression levels of TSPAN8 (left panel) and p53 (right panel) transcripts were assessed by reverse transcriptase (RT)–quantitative PCR (QPCR) in non-invasive IC8 and invasive T1C3 melanoma cells, previously described in Berthier-Vergnes *et al.*^1^ (*n*=3; ±s.e.m.). We checked that the TP53 gene was wild-type in these melanoma cell lines. The RT-QPCR protocol and primers used were described in Agaesse *et al.*,^14^ except for p53 primers: p53-forward 5′-TGACTGTACCACCATCCACTA-3′ and p53-reverse 5′-AAACACGCACCTCAAAGC-3′. Statistical significance was calculated by a two-tailed Student's *t*-test for unpaired samples. Mean differences were considered significant when *P*<0.05 and ***P<0.001. NS, nonsignificant. (**c**) Expression levels of Tspan8 and p53 proteins were assessed by western blot in non-invasive IC8 and invasive T1C3 melanoma cells (*n*=3; a representative experiment is shown). Western blots were performed as previously described in.^14^ β-Actin (clone C4 Millipore 1/5000, Darmstadt, Germany) was used as a loading control. Tspan8 was detected using a mouse monoclonal anti-Tspan8 antibody (TS29.2 clone 1/2000)^38^ and p53 using a mouse monoclonal anti-p53 antibody (D01, Santa Cruz, Santa Cruz, CA, USA).

Ensembl.org describes three coding isoforms in humans, which all encode the same Tspan8 protein of 237 amino acids. We previously showed that invasive melanoma cells predominantly express isoform 2, which is transcribed from an internal promoter.^14^ *In silico* analysis of this promoter (pTSPAN8) by the MatInspector gene regulation software (Genomatix.de) revealed that pTSPAN8 contains consensus-binding sites for many transcription factors, and in particular two repressor half-sites for the p53 tumor-suppressor gene (with consensus sequence RRXCXXGXYX/XRXCXXGXYY) that are located in the second exon of TSPAN8 full-length complementary DNA (Figure 1a). As p53 is a tumor repressor^7^ and Tspan8 a pro-metastatic tetraspanin, we postulated that p53 could be a repressor of TSPAN8 expression in melanoma cells. We first assessed whether a decrease in baseline p53 expression between non-invasive (IC8 clone) and invasive (T1C3 clone) melanoma cells, two clones in which TP53 gene is wild-type, could explain the occurrence of Tspan8 expression in the latter (Figure 1b, left panel and Figure 1c). Similar amounts of p53 were observed at both at mRNA (Figure 1b, right panel) and protein (Figure 1c) levels. This is consistent with the function of transcription factors, for which activation status is often much more important than expression level. We therefore investigated whether p53 exerts a negative regulatory role on Tspan8 expression by performing silencing experiments in various melanoma cell lines.

### p53 is a direct transcriptional repressor of Tspan8 expression

**Figure 2:**
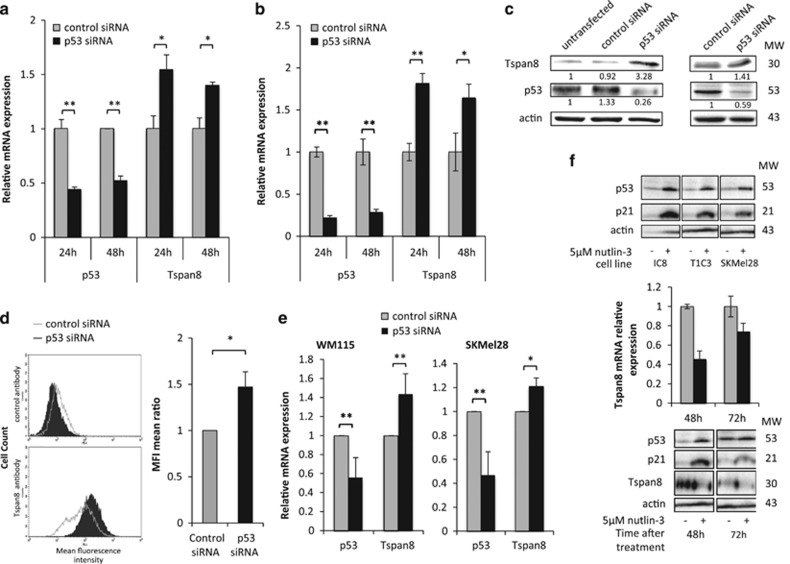
p53 silencing in non-invasive and invasive melanoma cells increases Tspan8 expression. (**a**, **b**) Quantitative PCR (QPCR) analysis showed p53 and TSPAN8 transcript expression levels 48 and 72 h after control or p53 small interfering RNA (siRNA) transfection in (**a**) non-invasive IC8 cells and (**b**) invasive T1C3 cells (*n*=3; ±s.d.). For siRNA transfection, 10^5^ cells per well were seeded in six-well plates and, after 24 h, transfected with 20 nM of control siRNA or p53 siRNA with INTERFERin (Polyplus, Illkirch, France). Targeting sequences were 5′-UAAGGCUAUGAAGAGAUAC-3′ for control siRNA and 5′-UAUGGCGGGAGGUAGACUG-3′ for p53 siRNA. (**c**) Expression levels of Tspan8 and p53 proteins were assessed by western blot in non-invasive IC8 (upper panel) and invasive T1C3 (lower panel) melanoma cells (*n*=3; a representative experiment is shown). Western blot quantifications were performed using ImageJ software (NIH/ImageJ, Bethesda, MD, USA). (**d**) Mean Tspan8 cell surface protein expression was assessed by fluorescence-activated cell sorting cytometry (FACS) in invasive T1C3 melanoma cells 72 h post-transfection with control or p53 siRNA, as previously described in Berthier-Vergnes *et al.*^1^ The left panel is representative of three independent experiments and the right panel represents the mean±s.d. of three independent experiments. (**e**) QPCR analysis showed p53 and TSPAN8 transcript expression levels 48 to 72 h after control or p53 siRNA transfection in non-invasive WM115 (left panel) and invasive SKMel28 (right panel) cells, in which TP53 gene is not mutated (*n*=3; ±s.d.). (**f**) The effect of nutlin-3 (5 μM; N-6287 Sigma-Aldrich, St Louis, MO, USA) on p53, p21 and Tspan8 expression in invasive T1C3 melanoma cells was assessed at 48 and 72 h post-treatment compared with control vehicle treatment (dimethylsulfoxide (DMSO)). Tspan8 mRNA levels (middle panel) were measured by QPCR (*n*=3; ±s.d.) and protein expression levels (upper and lower panel) of p53, p21 and Tspan8 were assessed by western blot (*n*=2; a representative experiment is shown). Statistical significance was assessed by two-tailed Student's *t*-test for unpaired samples. Mean differences were considered significant when *P*<0.05, *P<0.05 and **P<0.01.

We assessed the effect of p53 inhibition on Tspan8 expression. Using small interfering RNA, we showed that efficient p53 silencing in human non-invasive IC8 melanoma cells is sufficient to turn on Tspan8 expression at mRNA (Figure 2a) and protein (Figure 2c, upper panel) levels. This significant increase in Tspan8 expression was also observed in another non-invasive melanoma cell line, WM115 (Figure 2e, left panel). In invasive T1C3 cells, Tspan8 was expressed at high baseline level and p53 silencing further increased expression, at both mRNA (Figure 2b) and protein (Figure 2c, lower panel) levels. The overexpressed Tspan8 protein was even addressed to the membrane, as it was detected at cell surface by flow cytometry analysis (Figure 2d). Tspan8 expression was also increased in another cell line, SKMel28, known for invasiveness (Figure 2e, right panel). Overall, these findings showed that p53 silencing is sufficient to induce Tspan8 expression. To confirm these conclusions, we performed gain-of-function by inducing stabilization of p53 using Nutlin-3, an inhibitor of p53 degradation (Figure 2f). This experiment corroborated that all cell lines used in this study responded to p53 activation, as cell treatment with 5 μM Nutlin-3 during 48 h induced p21 mRNA expression by 7.7-, 10.8-, 5.4- and 11-fold in IC8, T1C3, SKMel28 and WM115, respectively (data not shown), and stabilized p53 and p21 protein expression (Figure 2f). As expected, p53 stabilization in invasive melanoma cells is sufficient to decrease Tspan8 expression at mRNA and protein levels (Figure 2f). Unlike many types of human cancer, in which TP53 gene is often mutated,^15^ TP53 is intact in >95% of human melanomas (for a review, see Chin^16^). However, it was recently shown that wild-type p53 was transcriptionally inactive in several melanoma cell lines^17^ and that p53 had a role in melanomagenesis in several animal models.^18, 19, 20, 21, 22, 23^ It can thus be postulated that, in non-invasive melanoma cells, p53 could act to repress Tspan8 expression, and that a decrease in p53 repression activity triggers Tspan8 expression. This is consistent with the repressive role of p53 on several other target genes (for a review, see Riley *et al.*^24^).

**Figure 3:**
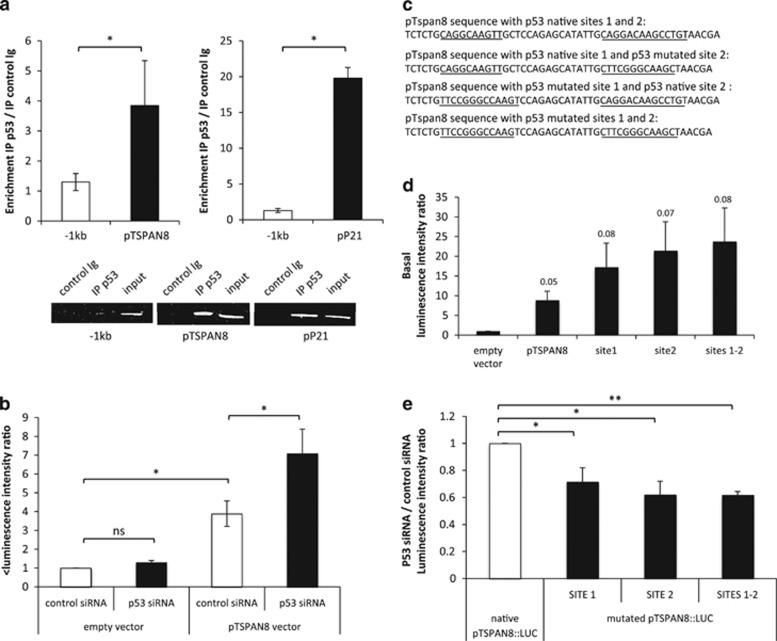
p53 is recruited onto p53 consensus-binding sites in TSPAN8 promoter and represses Tspan8 expression. (**a**) p53-Chromatin immunoprecipitation (ChIP) assays were performed in invasive T1C3 melanoma cells (*n*=4). Enrichment of TSPAN8 promoter region (left panel) was analyzed in comparison with a negative control promoter region located −1-kb upstream of the beginning of TSPAN8 promoter. p21 Promoter region (right panel) was used as positive control. ChIP experiments were performed, as previously described by Masse *et al.*^39^ A representative experiment after agarose gel electrophoresis is shown in the lower panel. (**b**–**e**) Luciferase assays were performed with 20 nM of control or p53 small interfering RNA (siRNA) combined with 250 ng of (**b**) native pTSPAN8::LUC (constructed as described in Agaesse *et al.*^14^) and/or (**d**, **e**) pTSPAN8 in which p53 consensus-binding sites were mutated. Directed mutagenesis was performed using the In Fusion HD Cloning Plus kit (Ozyme, Saint-Quentin-en-Yvelines, France), according to the manufacturer's instructions. PCR was performed at 55 °C. Primers used for mutagenesis were: forward 5′-TTCCGGGCCAAGTCCAGAGCATATTGCAGGA-3′ and reverse 5′-CTTGGCCCGGAACAGAGATTTCTGTATCCACG-3′ for ‘site 1’ forward 5′-CTTCGGGCAAGCTAACGAATAGTTAAATTCACGGC-3′ and reverse 5′-GCTTGCCCGAAGGCAATATGCTCTGGAGCA-3′ for ‘site 2’, and final sequences were described in **c**. For luciferase experiments, 10^5^ cells per well were seeded in 24-well plates and, after 24 h, transfected with 250 ng of control plasmid or plasmid of interest for 24 h, combined with 20 nM of control siRNA or p53 siRNA. Luciferase assays were performed using the Dual-Luciferase Reporter Assay System (Promega, Madison, WI, USA) according to the manufacturer's instructions. The luminescence intensity ratio was calculated relative to that of the pGL4.10-empty-vector. Data were normalized to the transfection efficacy assessed by pCMV-RL vector co-transfection. At least three independent biological replicates were performed. Statistical significance was assessed by two-tailed Student's *t*-test for unpaired samples. Mean differences were considered significant when *P*<0.05, *P<0.05 and **P<0.01. NS, nonsignificant.

We then investigated whether p53 exerts its effect on TSPAN8 expression directly, through the putative half-p53 consensus-binding sites we identified in pTSPAN8. We first performed chromatin immunoprecipitation experiments using a p53 antibody versus a control immunoglobulin, and used quantitative PCR to analyze p53 enrichment on pTSPAN8 (Figure 3a). We showed that, compared with a negative control located −1- kb upstream of pTSPAN8, p53 was enriched 20-fold on the positive control p21 promoter and significantly enriched fourfold on p53 consensus sites of TSPAN8 promoter. This indicates that endogenous p53 is specifically recruited onto p53 sites of pTSPAN8 in melanoma cells. We then tested whether TSPAN8 transcription activation was dependent on p53 consensus-binding sites in pTSPAN8, by performing luciferase assays on pTSPAN8 containing native or mutant p53-binding sites. As previously described,^14^ the promoting sequence of TSPAN8 significantly increased luciferase activity 3.8-fold compared with empty vector. We showed that inhibiting p53 by small interfering RNA had no impact on the control empty vector but was sufficient to further significantly increase luciferase activity sevenfold under the control of pTSPAN8 (Figure 3b). Moreover, when one or both half-p53 consensus-binding sites of pTSPAN8 were mutated (Figure 3c), basal Tspan8 promoter repression was partially lost (Figure 3d) and p53 silencing did not enhance luciferase intensity as strongly as did native pTSPAN8 (Figure 3e), demonstrating that p53 is a direct transcriptional repressor of TSPAN8, through p53-binding sites in pTSPAN8.

To sum up, the data showed that TSPAN8 expression could be turned on in melanoma cells when its direct transcriptional regulator, p53, is silenced. Several mechanisms have been proposed recently for p53 inactivation in melanoma. MDM4 overexpression may promote metastatic progression by antagonizing p53's pro-apoptotic function.^25^ MDM2, cyclin B1 and nuclear iASPP are also enriched in melanoma metastasis and may lead to p53 inhibition.^26^ p53 may also be deactivated through CDKN2A deletion or mutation, as occurs in around 40% of melanoma cases.^27^ Downregulation of miR-18 or miR-3151, combined with BRAF inhibition, may result in regulation either of the MDM2-p53 axis^26^ or of the nuclear p53 location,^28^ respectively. Hence, considering the role we previously described for Tspan8,^1, 14^ we can hypothesize that an inactivation of p53, by any of these mechanisms, may induce TSPAN8 expression and acquisition of an invasive phenotype in melanoma.

### p53 regulates *in vitro* melanoma cell invasion in a Tspan8-dependent manner

**Figure 4:**
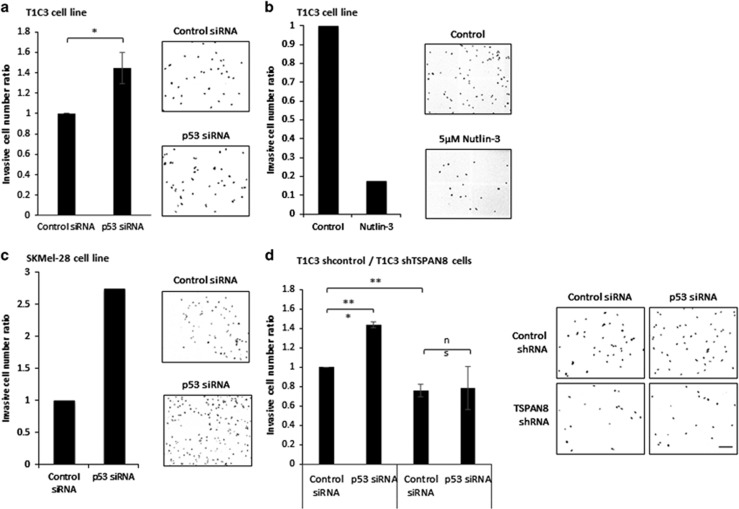
p53 modulates matrigel invasion in invasive cells in a Tspan8-dependent manner. (**a**–**d**) Boyden chamber matrigel invasion assays were performed on control or p53 small interfering RNA (siRNA) transfected invasive T1C3 (**a**) and SKMel28 (**c**) cells, on invasive T1C3 cells treated 48 h with 5 μM Nutlin-3 or dimethylsulfoxide (DMSO; control condition) (**b**) and on T1C3 cells stably expressing either control or TSPAN8 short hairpin RNA (shRNA) (**d**). In all, 10^5^ cells per well were seeded in six-well plates. Transfections or Nutlin-3 treatment were performed 24 h later with 20 nM of control or p53 siRNA or 5 μM Nutlin-3 or DMSO. After 36 h, cells were resuspended in 10% fetal calf serum (FCS)–McCoy’s medium by trypsination and centrifuged at 1200* g* for 5 min. The residual FCS of the pellet was washed in 1 ml of phosphate-buffered saline and centrifuged. Finally, pelleted cells were resuspended in McCoy’s medium without FCS and for each condition, 35 000 cells were seeded in McCoy’s medium without FCS in the upper Boyden’s chamber, whereas the lower chamber was loaded with 5 μg/ml−10% FCS–McCoy’s medium. After 36 h of incubation, all the matrigel was scrubbed away, cells were fixated on the membrane for 15 min in −20 °C methanol, and stained for 10 min in 1 mM 4,6-diamidino-2-phenylindole (DAPI). The whole membrane surface was scanned at a magnification of × 100 using a time-lapse microscope scan slide protocol (ZEISS, Oberkochen, Germany), and all invaded cells were counted. Two (**b**, **c**; a representative experiment was shown) or three (**a**, **d**) independent biological replicates were performed. A representative visual field is illustrated in the right panel; scale bar=200 μM. Statistical significance was calculated by two-tailed Student's *t*-test for unpaired samples. Mean differences were considered significant when *P*<0.05, *P<0.05 and **P<0.01. NS, nonsignificant.

We have previously shown that Tspan8 promoted melanoma invasion through matrigel without interfering with proliferation or migration behavior.^1^ We therefore hypothesized that Tspan8 expression regulated by p53 would not affect the cell cycle as previously described in mouse melanoma,^22^ but would rather regulate cell invasion. Recent studies demonstrated that wild-type p53 modulates the invasiveness of breast cancer cells,^29^,^30^ osteosarcoma cells^31^ and hepatocellular carcinoma cells.^32^ In melanoma, very little is known about the involvement of p53 in invasion. One study reported that expression of a dominant-negative form of p53 strongly increased the invasiveness of A375P melanoma cells, whereas wild-type p53 inhibited it.^33^ Very recently, Roth *et al.* showed that the mouse-specific isoform Δ122p53, similar to the human Δ133p53 isoform, which is overexpressed in melanoma,^34^ promoted invasion in an IL-6- and CCL2-dependent manner.^35^ We therefore tested whether p53 could specifically regulate invasion in a Tspan8-dependent manner. p53 silencing was shown to increase the invasiveness of T1C3 melanoma cells in Boyden chambers (Figure 4a), whereas p53 stabilization by nutlin-3 treatment is sufficient to decrease invasive capacities (Figure 4b). p53 silencing also enhanced invasive capacities of SKMel28 invasive cells (Figure 4c). To determine whether p53 affected melanoma invasion through Tspan8 regulation, epistasis experiments were performed, using T1C3 cells in which TSPAN8 expression was efficiently silenced after selection of cells stably expressing TSPAN8 short hairpin RNA (T1C3/shTSPAN8).^14^ The results showed that T1C3/shTSPAN8 cells were less invasive than T1C3/shcontrol cells, confirming that TSPAN8 expression promotes invasiveness in matrigel. Moreover, inhibiting p53 in T1C3/shcontrol cells increased the number of invading cells, and this increase was partially abolished by inhibiting p53 expression in T1C3/shTSPAN8 (Figure 4d).

In conclusion, we demonstrated that p53 represses melanoma invasiveness *in vitro* in a Tspan8-dependent manner. It can thus be hypothesized that inactivation of p53 transcriptional repressor activity triggers Tspan8 expression and subsequent acquisition of invasiveness. This study highlights that reactivating p53 could be a therapeutic strategy in melanoma. However, although this strategy was already proposed and showed promising results, for example using MDM4,^25^ MDM2 or iASPP^36^ inhibitors, it was hitherto thought that p53 reactivation would act mainly on p53-dependent apoptosis and cell cycle arrest functions. Indeed, it was shown that p53 reactivation by PRIMA-1^Met^ synergizes with BRAF inhibition by vemurafenib to induce apoptosis and suppress proliferation of BRAF mutant melanoma cells *in vitro*.^37^ As we previously showed that Tspan8 expression is also decreased by vemurafenib,^14^ it can now be hypothesized that reactivating p53 and inhibiting BRAF signaling would help to decrease melanoma cells' invasiveness by repressing Tspan8 expression, and could be promising in future melanoma therapies.
